# EXPLORING THE FEASIBILITY AND UTILIZATION OF CLARE: A MOBILE APPLICATION FOR CAREGIVERS AFTER HOSPITAL DISCHARGE

**DOI:** 10.1093/geroni/igae098.2145

**Published:** 2024-12-31

**Authors:** Dingyue Wang, Doreen Matters, Cristina Hendrix

**Affiliations:** Duke University, Seattle, Washington, United States; Duke University, Durham, North Carolina, United States; Duke University, Durham, North Carolina, United States

## Abstract

CLARE is the first mobile application tailored to enhance discharge education and teaching practices in hospital settings. This mixed-method study aimed to assess the feasibility of CLARE in providing caregiving training and support immediately following hospital discharge. Additionally, the study sought to apply the Unified Theory of Acceptance and Use of Technology (UTAUT) framework to identify factors that promote or hinder the intent and use of CLARE among caregivers. Caregivers included in the study were primary caregivers assisting patients post-discharge, aged at least 35 years, and caring for patients aged at least 55 years. Data from application usage was analyzed alongside caregiver interviews. CLARE areas of teaching encompassed discharge preparation (e.g., early mobility instruction), COVID-19 care protocols (e.g., isolation; quarantine; home sanitization; monitoring shortness of breath), and caregiver self-care strategies (e.g., managing anxiety and stress). Preliminary findings from the 23 enrolled caregivers (with a target enrollment of 50) revealed that 65.2% were female, and the majority (78.3%) were between 50 and 80 years old. Usage statistics indicated that 17.4% of caregivers completed all tasks, 34.8% completed over 80% of tasks, and 52.2% completed more than half of the tasks in CLARE. Survey and interview responses showed that CLARE is a user-friendly, time-efficient, and informative app that facilitates awareness of caregiver needs. Caregivers appreciated the clear and real-time information in CLARE. Our findings suggest a need for ongoing development and updates to CLARE, alongside comprehensive interventions addressing the diverse hospital discharge needs of caregivers.

